# Preparation and Epitope Identification of Monoclonal Antibodies against the NS6 Protein of Porcine Deltacoronavirus (PDCoV)

**DOI:** 10.3390/ijms25147645

**Published:** 2024-07-12

**Authors:** Huiguang Wu, Xian Sun, Chen Li, Sihan Xie, Zhenhai Chen

**Affiliations:** 1 College of Veterinary Medicine, Yangzhou University, Yangzhou 225009, China; 2 Joint International Research Laboratory of Agriculture and Agri-Product Safety, Ministry of Education of China, Yangzhou University, Yangzhou 225009, China; 3 Jiangsu Co-Innovation Center for Prevention and Control of Important Animal Infectious Diseases and Zoonoses, Yangzhou University, Yangzhou 225009, China

**Keywords:** porcine deltacoronavirus, NS6 protein, monoclonal antibody, antigenic epitope

## Abstract

Porcine deltacoronavirus (PDCoV) is an emerging enteric pathogen that causes substantial economic losses in the swine industry worldwide. The PDCoV NS6 protein is an accessory protein that plays a pivotal role in the viral life cycle and immune evasion. However, the functions of NS6 and its role in PDCoV pathogenesis remain largely unknown. In this study, we prepared a monoclonal antibody (mAb) 5-A11 that specifically recognizes the PDCoV NS6 protein. The mAb 5-A11 exhibited high specificity for PDCoV, with no cross-reactivity with several major porcine pathogenic viruses. Furthermore, the epitope recognized by mAb 5-A11 was precisely mapped to residues ^70^EYGSIYGKDFI^80^ of the NS6 protein using Western blot analysis. Notably, this epitope is highly conserved among different PDCoV isolates. Substantial variations were observed when comparing this epitope with the corresponding regions in the NS6 proteins of other δ coronaviruses, suggesting potential differences in the structure, function, and antigenicity of their NS6 proteins. Our findings provide valuable tools and insights for further elucidating the functions of the NS6 protein and its role in PDCoV pathogenesis, as well as for developing diagnostic and therapeutic strategies against PDCoV infection.

## 1. Introduction

*Deltacoronavirus suis* (porcine coronavirus HKU15, commonly known as porcine deltacoronavirus or PDCoV) is an emerging enteropathogenic coronavirus that causes substantial morbidity and mortality in swine, resulting in a significant economic burden on the global pork industry. PDCoV was first identified in Hong Kong in 2012 [1] and subsequently gained prominence following a notable outbreak in OH, USA, in 2014 [2]. Since its initial identification in the United States, PDCoV has rapidly disseminated throughout the country and has been documented in numerous countries worldwide, including Canada [3], Mexico [4], Peru [5], South Korea [6], China [7], Japan [8], Thailand [9], Laos [10], Vietnam [11], and several other Southeast Asian countries. The global spread of PDCoV underscores its high transmissibility and highlights the swine industry’s vulnerability to emerging infectious diseases.

In swine populations, the virus primarily spreads through the fecal–oral route, a process facilitated by its stability in the environment and the high-density living conditions of commercial pig farms [12]. The enteric transmission route of this virus, which causes severe diarrhea and vomiting in infected swine, is characterized by viral shedding in the feces, leading to the contamination of feed, water, and fomites, thereby facilitating efficient viral dissemination and rapid spread through susceptible swine herds [9]. Moreover, studies suggest that PDCoV can be transmitted through aerosols, which are inhaled when viral particles are present in the air, especially in densely populated settings such as swine farms, thereby enhancing the virus’s spread over distances [13]. These findings highlight the importance of gaining a comprehensive understanding of PDCoV transmission dynamics to develop effective control measures.

In addition to transmission among pigs, there is a serious concern that PDCoV possesses the potential for cross-species transmission. PDCoV has demonstrated a notable ability to infect a wide range of hosts, including avian species [1,14], pigs [2], mice [15], and cattle [16]. The virus can infect a variety of cell types in vitro [14,17,18], including human and avian cells, suggesting the potential for zoonotic and reverse zoonotic events. PDCoV utilizes the aminopeptidase N protein as an entry co-factor for cell entry via an endocytotic pathway [17,19,20,21], thereby facilitating infection across different species. Recent findings indicate that PDCoV cell-to-cell transmission is resistant to neutralizing antibodies and immune sera that potently neutralize free viruses [22]. The capacity of PDCoV to disseminate across species can be attributed to its extensive host range, frequent host switching events, and genetic recombination. Notably, cases of PDCoV infection have been documented in children [23], underscoring the necessity for continued surveillance and research to elucidate the underlying mechanisms of its evolution and interspecies transmission.

PDCoV NS6 is an accessory protein that plays a crucial role in the viral life cycle and immune evasion. NS6 is primarily located in the cytoplasm and co-localizes with the endoplasmic reticulum (ER) and ER-Golgi intermediate compartments, suggesting its potential roles in viral assembly and intracellular trafficking [24]. Notably, NS6 significantly inhibits Sendai virus-induced interferon-beta (IFN-β) production by interfering with the binding of RIG-I/MDA5 to double-stranded RNA (dsRNA). This inhibition reduces RIG-I-like receptor (RLR)-mediated IFN-β production, allowing NS6 to function as an effective interferon antagonist [25]. Moreover, NS6 interacts with the VPS35 retromer component to modulate intracellular vesicle trafficking, demonstrating its role in hijacking host cellular pathways for viral benefit [26]. A recent study has shown that NS6 is expressed during in vivo infection and incorporated into PDCoV virions, suggesting its dual role as both an accessory and structural protein [27]. The multifaceted roles of NS6 in modulating host immune responses, facilitating viral replication, and its incorporation into virions underscore its critical role in PDCoV pathogenesis, offering valuable insights for the development of targeted antiviral strategies.

Monoclonal antibodies (mAbs) offer targeted diagnostic capabilities by specifically recognizing and binding to unique antigens, enabling the identification and neutralization of specific pathogens or the marking of affected cells. Extensive research on mAbs against PDCoV has been conducted, resulting in a deeper understanding of the virus’s proteins, and facilitating the development of diagnostic innovations. For example, mAbs targeting the membrane (M) protein, such as mAb 24-A6, have demonstrated high specificity for PDCoV without cross-reactivity to several major porcine viruses, thereby highlighting their diagnostic potential [28]. Efforts to characterize other proteins like the nucleocapsid (N) [29,30,31,32,33,34,35,36], spike (S) [37], NS6 [24,38], and NS7 [39] have also yielded mAbs that accurately differentiate PDCoV from other coronaviruses. Nevertheless, despite these advances, challenges such as the insufficient specificity of certain mAbs and their sensitivity to viral mutations have been identified, which may potentially limit their effectiveness in practical applications. These limitations underscore the critical need to expand our research to include mAbs against additional PDCoV proteins to enhance both the reliability and the applicability of these diagnostic tools.

The objective of the present study focused on preparing mAb against the PDCoV NS6 protein using an *Escherichia coli* (*E. coli*) expression system and characterizing the properties of the mAb. By immunizing mice with the recombinant protein, we established a hybridoma cell line that secretes mAbs specific to the PDCoV NS6 protein. The specificity of the mAbs was rigorously evaluated using techniques such as indirect immunofluorescence assay (IFA) and Western blot analysis. The linear epitope recognized by the mAbs was precisely identified through truncation mapping. Our study contributes to a deeper understanding of the immunogenic properties of PDCoV and facilitates the development of targeted diagnostic and therapeutic strategies specifically tailored for PDCoV infections.

## 2. Results

### 2.1. Plasmid Construction and Protein Expression

**Figure 1:**
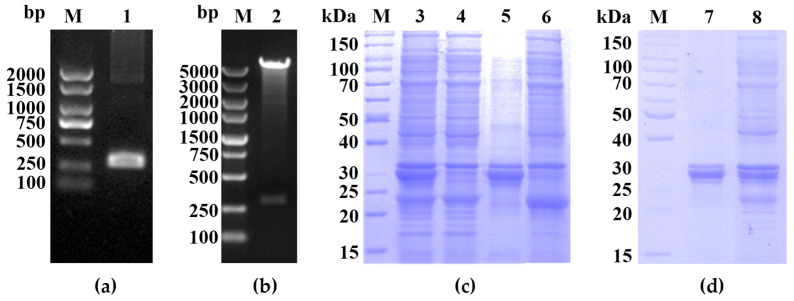
Expression and purification of the recombinant NS6 protein. (**a**) Amplified product of the *NS6* gene. Lane M, DL2000 DNA maker; lane 1, PCR product of the *NS6* gene. (**b**) Identification of recombinant plasmids (pGEX-6p-1-NS6) by enzymatic digestion. Lane M, DL5000 DNA marker; lane 2, digested products of pGEX-6p-1-NS6. (**c**) Prokaryotic expression of recombinant GST-NS6 fusion protein. Lane M, protein molecular weight marker (cat# 26614); lane 3, whole cell lysate of *E. coli* BL21 transformed with empty vector PGEX-6p-1; lanes 4, 5, and 6, whole cell lysate, supernatant, and pellet, respectively, of *E. coli* BL21 transformed with PGEX-6P-1-NS6 (induced with IPTG). (**d**) Purification of the recombinant GST-NS6 fusion protein. Lane M, protein molecular weight marker (cat# 26614); lane 7, purified GST-NS6 fusion protein from PGEX-6p-1-NS6; lane 8, whole cell lysate of *E. coli* BL21 transformed with PGEX-6p-1 (induced with IPTG).

The *NS6* gene from the PDCoV CHN-GD16-05 isolate was amplified and inserted into the prokaryotic expression vector pGEX-6p-1 (Figure 1a). The correct construction of the recombinant plasmid was confirmed by restriction endonuclease digestion and agarose gel electrophoresis (Figure 1b). 

Sodium dodecyl sulfate–polyacrylamide gel electrophoresis (SDS-PAGE) analysis revealed a distinct band at approximately 35 kDa, consistent with the expected size of the GST-NS6 fusion protein (Figure 1c). The target protein was extracted from the gel and purified through a series of steps, including protein electrophoresis elution, salt ion balance, and concentration. These steps ultimately yielded the purified recombinant GST-NS6 fusion protein (Figure 1d).

### 2.2. IFA and Western Blot Identification of mAb 5A-11 against PDCoV NS6 Protein

**Figure 2:**
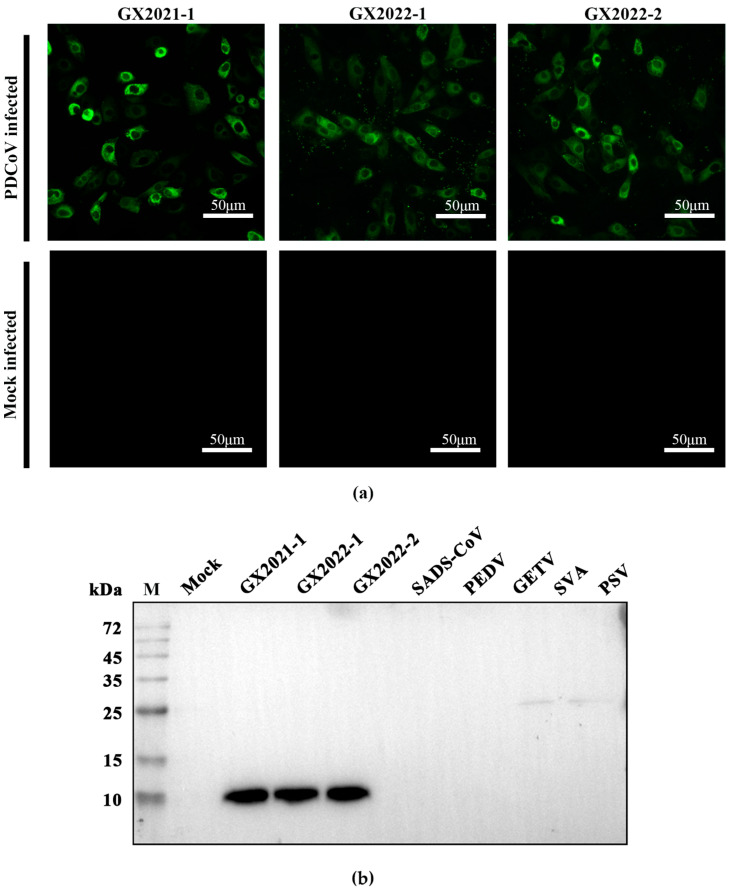
Identification of mAb against PDCoV NS6 protein. (**a**) Immunofluorescence assay (IFA) detection of PDCoV-infected cells using mAb 5-A11. The green fluorescence represents the binding of mAb 5-A11 to NS6 protein in cells infected with different isolates of PDCoV. (**b**) Western blot analysis of mAb 5-A11 reactivity. The specificity of mAb 5-A11 was evaluated by Western blotting using cell lysates from cells infected with PDCoV isolates (GX2021-1, GX2022-1, and GX2022-2), SADS-CoV, PEDV, GETV, SVA, and PSV.

LLC-PK1 cells were infected with three PDCoV isolates (GX2021-1, GX2022-1, and GX2022-2), and mAb 5-A11 was used as the primary antibody for immunofluorescence detection. IFA results showed that mAb 5-A11 reacted with all three PDCoV-infected cell cultures (Figure 2a), confirming its ability to detect multiple PDCoV isolates. Western blot analysis was performed using mAb 5-A11 as the primary antibody on cell lysates from LLC-PK1 cells infected with the three PDCoV isolates and ST cells infected with swine acute diarrhea syndrome coronavirus (SADS-CoV), porcine epidemic diarrhea virus (PEDV), Getah virus (GETV), Seneca virus A (SVA), and porcine sapelovirus (PSV). A specific target protein band (approximately 35 kDa) was detected in the lanes corresponding to all three PDCoV-infected LLC-PK1 cell lysates (Figure 2b). In contrast, no specific bands were detected in the lanes corresponding to SADS-CoV, PEDV, GETV, SVA, and PSV-infected ST cell lysates, indicating the high specificity of mAb 5-A11 for PDCoV.

### 2.3. Epitope Mapping of PDCoV NS6 Protein

**Figure 3:**
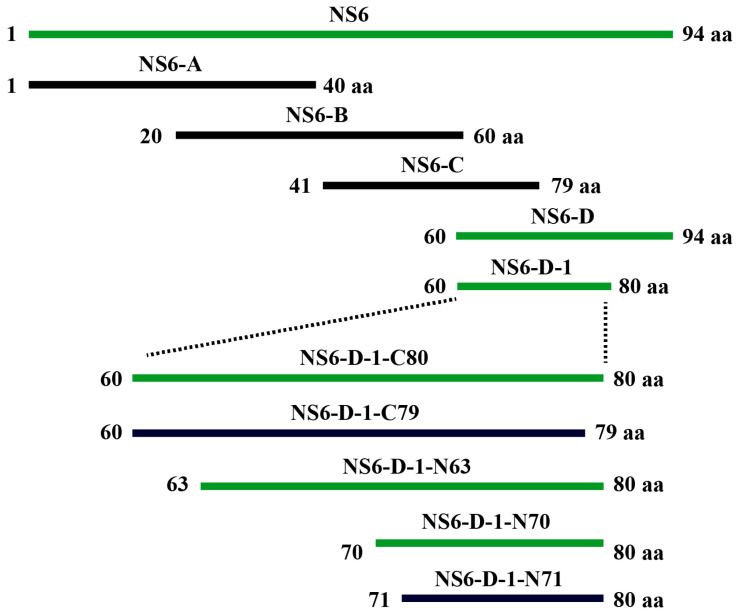
Schematic representation of PDCoV NS6 protein truncations used for B-cell epitope mapping. Green fragments indicate peptides that reacted with mAb 5-A11, while black fragments represent peptides that did not react with mAb 5-A11.

**Figure 4:**
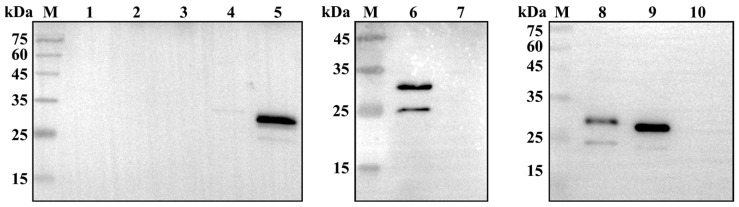
Identification of the B-cell epitope of the PDCoV NS6 protein. A series of truncated fragments were cloned into the plasmid pEGFP-C3 and expressed in HEK293A cells. Ten peptides were expressed to detect the minimal epitope and tested for reactivity with mAb 5-A11 by Western blotting. Lane M, tricolor pre-dyed protein molecular weight marker; lane 1, empty plasmid pEGFP-C3 (control); lanes 2–10, peptides NS6-A (1–40 aa), NS6-B (20–60 aa), NS6-C (41–79 aa), NS6-D (60–94 aa), NS6-D-1 (60–80 aa), NS6-D-1-C79 (60–79 aa), NS6-D-1-N63 (63–80 aa), NS6-D-1-N70 (70–80 aa), and NS6-D-1-N71 (71–80 aa).

To identify the specific epitope on the PDCoV NS6 protein recognized by mAb 5-A11, the amino acid sequence of the NS6 protein (94 amino acids) was divided into a series of overlapping peptide fragments (Figure 3). Western blot analysis showed that mAb 5-A11 recognized the peptide segment NS6-D (amino acids 60–94) (Figure 4), indicating that the epitope recognized by mAb 5-A11 is located within this region of the PDCoV NS6 protein. Further truncation analysis narrowed down the epitope to a smaller segment, NS6-D-1 (amino acids 60–80), which was also recognized by mAb 5-A11 in Western blot (Figure 4). Sequential truncation and expression analysis ultimately mapped the epitope recognized by mAb 5-A11 on the PDCoV NS6 protein to the peptide sequence ^70^EYGSIYGKDFI^80^ (Figure 4).

### 2.4. Spatial Localization and Conservation of the Epitope

**Figure 5:**
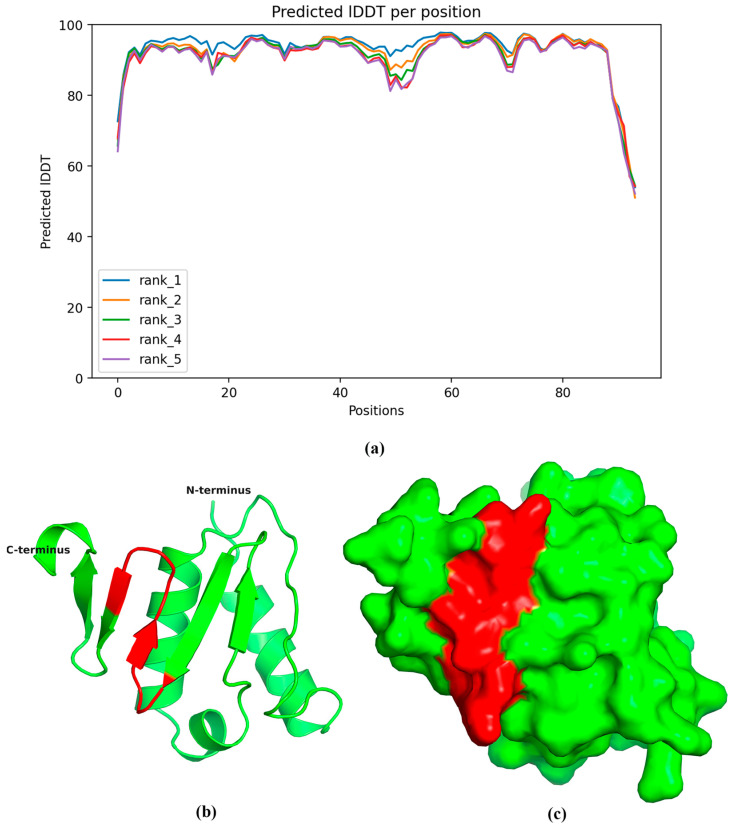
Predicted three-dimensional (3D) structure of the PDCoV NS6 protein. (**a**) The predicted Local Distance Difference Test (pLDDT) value over 94 amino acid residues of the PDCoV NS6 protein. The higher values indicate greater model confidence. (**b**) Cartoon representation of the predicted NS6 protein structure. (**c**) Surface representation of the predicted NS6 protein structure. The antigenic epitope ^70^EYGSIYGKDFI^80^ recognized by mAb 5-A11 is marked in red.

The three-dimensional structure of the PDCoV NS6 protein was predicted using AlphaFold2 (version 2.3.2) and visualized using PyMOL (open-source version 2.5.0). The predicted Local Distance Difference Test (pLDDT) scores for the majority of amino acid residues were above 90 (Figure 5), indicating high confidence in the predicted structure. The predicted model suggests that the NS6 protein consists of two structural domains: an N-terminal alpha-helical domain (helices α1, α2, and α3) and a C-terminal β-sheet domain (strands β1–β5) (Figure 5). According to the predicted model, the antigenic epitope ^70^EYGSIYGKDFI^80^ is located in the β-fold domain, specifically within β2-β4 of the NS6 protein (Figure 5). 

**Figure 6:**
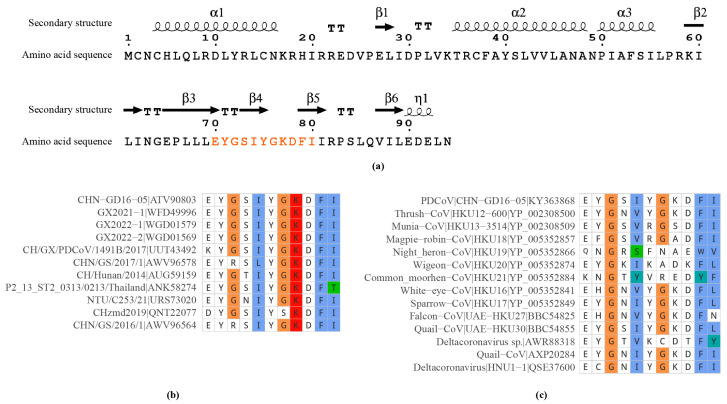
Sequence analysis of PDCoV NS6 protein and mAb 5-A11 epitope variation across coronavirus isolates. (**a**) The amino acid sequence and predicted secondary structure of the PDCoV NS6 protein. α-helices and η-helices are displayed as squiggles, β-strands are rendered as arrows, and strict β-turns are displayed as “TT” letters in the predicted secondary structure. The epitope region recognized by mAb 5-A11 is highlighted in orange. (**b**) Sequence alignment of the mAb 5-A11 epitope region among representative PDCoV isolates. (**c**) Sequence alignment of the corresponding region in representative deltacoronavirus isolates.

We retrieved and aligned the amino acid sequences of the NS6 protein from all 202 PDCoV isolates available in the GenBank database as of April 2024. The alignment results revealed that 195 out of 202 PDCoV isolates (approximately 96.5%) shared identical amino acid sequences of the mAb 5-A11 epitope on the NS6 protein. In contrast, only seven isolates (approximately 3.5%) exhibited varying degrees of amino acid substitutions within the epitope (Figure 6). These substitutions were primarily concentrated in the first, third, fourth, fifth, seventh, and eleventh amino acid residues of the epitope sequence. These findings indicate that the ^70^EYGSIYGKDFI^80^ epitope on the PDCoV NS6 protein is highly conserved among PDCoV isolates. 

To assess the conservation of the mAb 5-A11 epitope across δ coronaviruses, we compared it with the corresponding sequences of the NS6 protein from 17 different δ coronavirus isolates using the InterPro (version 99.0) database and Conserved Domain Database (CDD; version 3.21). After removing redundant sequences, we found that the 14-remaining unique δ coronavirus isolate showed variations at different positions within the corresponding epitope sequence (Figure 6). These variations suggest that while the epitope ^70^EYGSIYGKDFI^80^ of mAb 5-A11 is conserved in PDCoV, it undergoes substantial alterations compared with other δ coronavirus isolates.

### 2.5. Sequence Analysis of mAb 5-A11 Light and Heavy Chains

**Figure 7:**
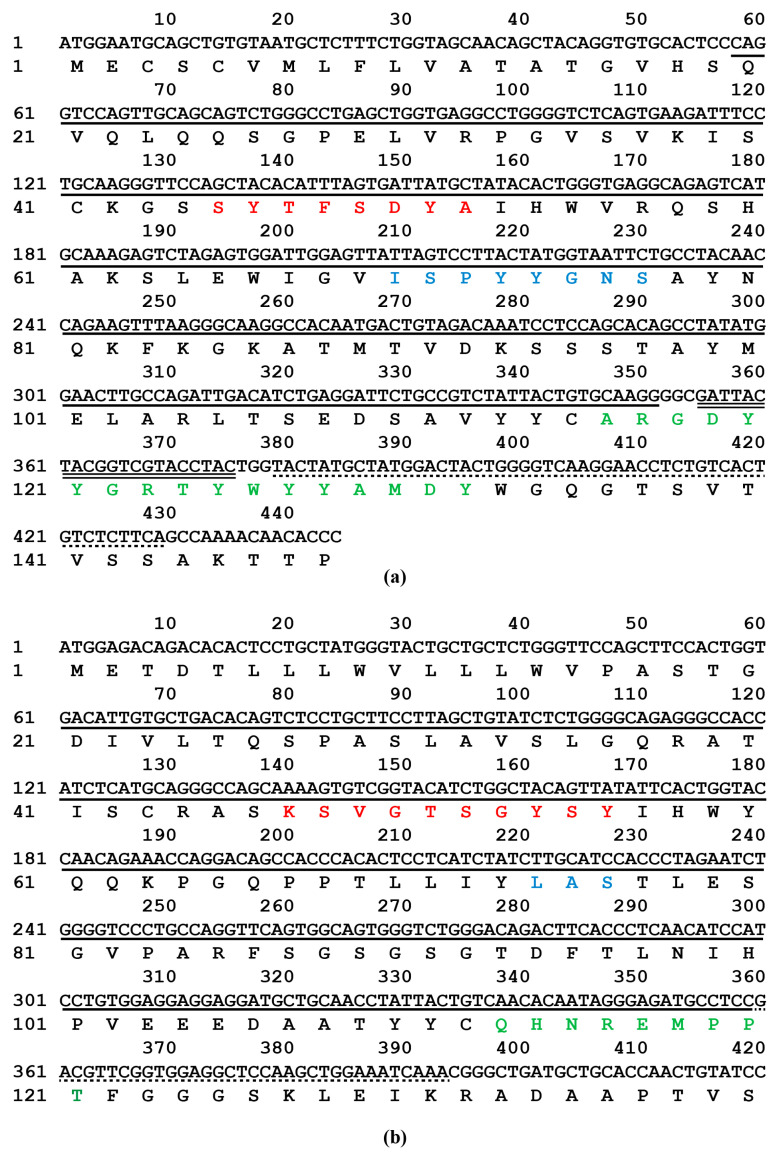
Nucleotide and deduced amino acid sequences of the variable regions of the heavy (**a**) and light (**b**) chains of mAb 5-A11. The complementarity-determining region 1 (CDR1), CDR2, and CDR3 are highlighted in red, blue, and green, respectively. Variable (V), diversity (D), and joining (J) regions are indicated by solid, double, and dotted lines, respectively.

IgBLAST analysis revealed that the light and heavy chain genes of mAb 5-A11 exhibited high similarity to mouse antibody genes, with 94.5% and 97.0% identity, respectively. Furthermore, the gene sequences of mAb 5-A11 aligned well with the mouse immunoglobulin (Ig) variable region framework structure. The V, D, and J segments of the mAb 5-A11 *VH* gene showed the highest similarity to the reference genes IGHV1-67*01 or IGHV1S137*01, IGHD1-1*01, and IGHJ4*01 in the international ImMunoGeneTics database (IMGT), respectively (Figure 7a). Likewise, the V and J segments of the mAb 5-A11 *VL* gene were most closely related to the reference genes IGKV3-12*01 and IGKJ1*01 in the IMGT database, respectively (Figure 7b). These findings suggest that the *VH* and *VL* genes of mAb 5-A11 were likely generated through V-(D)-J recombination of these germline gene segments.

## 3. Discussion

The PDCoV NS6 protein plays a crucial role in the viral life cycle and immune evasion. In this study, we prepared a monoclonal antibody designated 5-A11 that specifically recognizes the PDCoV NS6 protein with high specificity and does not cross-react with several major porcine pathogenic coronaviruses or porcine viruses. This antibody is a valuable tool for the diagnosis of PDCoV and further investigating the functions of the NS6 protein. The co-localization of NS6 with the endoplasmic reticulum (ER) and ER-Golgi intermediate compartments suggests its potential involvement in viral assembly and intracellular trafficking [24]. Moreover, NS6 has been demonstrated to inhibit interferon-beta (IFN-β) production by interfering with the binding of RIG-I/MDA5 to double-stranded RNA (dsRNA), thereby functioning as an effective interferon antagonist [25]. The interaction of NS6 with the VPS35 retromer component also indicates its role in modulating host cellular pathways for viral advantage [26]. The identification of the highly conserved ^70^EYGSIYGKDFI^80^ epitope on the NS6 protein in our study provides a potential target for further elucidating its functional domains and mechanisms of action in the context of PDCoV infection.

The selection of GST as an N-terminal fusion tag for the expression of the recombinant NS6 protein in *E. coli* raises the question of potential steric hindrance effects, given its relatively large size of approximately 26 kDa. Nevertheless, the expression and purification of GST-fusion proteins in *E. coli* expression systems have been extensively employed and validated, thus establishing them as a well-established and reliable strategy. The specific recognition of the near-native NS6 protein in PDCoV-infected cells by the prepared monoclonal antibody indicates that GST tag does not significantly affect the structure or antigenicity of NS6. Moreover, the predicted three-dimensional structure of NS6 indicates that the epitope recognized by the monoclonal antibody is located in the C-terminal region, distant from the N-terminal GST tag, suggesting a low likelihood of steric hindrance. These findings provide evidence to support the suitability of using the GST tag for the expression and purification of the NS6 protein in this study.

The epitope recognized by mAb 5-A11 against the NS6 protein was mapped to the sequence ^70^EYGSIYGKDFI^80^, which is located in the β-sheet region between β2-β4. The high degree of conservation of the epitope among different PDCoV isolates not only suggests its potential role in the structure, function, and evolutionary importance of the NS6 protein but also highlights its promise as a target for the development of PDCoV-specific diagnostic assays and vaccines. It is noteworthy that when the epitope was compared with the corresponding regions in the NS6 proteins of other δ coronaviruses, substantial sequence variations were observed. These alterations suggest potential differences in the structure, function, and antigenicity of their NS6 proteins, providing a new perspective for studying the molecular pathogenesis and evolutionary relationships of different δ coronaviruses.

Further research is necessary to address several limitations associated with the mAb 5-A11 against the NS6 protein. Firstly, the specific role of the ^70^EYGSIYGKDFI^80^ epitope in the function of the NS6 protein remains to be fully elucidated. Site-directed mutagenesis and functional analyses could help elucidate the functional importance of this epitope. Secondly, the present study focused on the preparation of monoclonal antibodies and epitope identification for the NS6 protein. Further research on the antigenic epitopes of other PDCoV proteins is necessary for a more comprehensive understanding of the virus. Finally, the practical diagnostic applications of mAb 5-A11 will require large-scale sample validation to determine its specificity and sensitivity. Future studies could also investigate the potential of this epitope as a target for the development of epitope-based vaccines to elicit protective immunity against PDCoV.

## 4. Materials and Methods

### 4.1. Viruses, Cells, and Animals

Three previously isolated and preserved PDCoV isolates, namely GX2021-1 (GenBank accession No. OQ547740), GX2022-1 (OQ736717), and GX2022-2 (OQ736716), were used in this study. Human embryonic kidney cells (HEK 293A), mouse myeloma cells (SP2/0), and porcine kidney epithelial cells (LLC-PK1) were maintained in Dulbecco’s Modified Eagle Medium (DMEM; Gibco, Grand Island, NY, USA) supplemented with 10% fetal bovine serum (FBS; Sigma-Aldrich, St. Louis, MO, USA). All cell lines were maintained at 37 °C in a humidified atmosphere containing 5% CO_2_. For conducting animal experiments, female BALB/c mice, aged six to eight weeks, were sourced from the Animal Experimental Center of Yangzhou University, China. The mice were housed in a specific pathogen-free (SPF) facility and provided with food and water ad libitum.

### 4.2. Expression and Purification of PDCoV NS6 Protein

The PDCoV *NS6* gene, derived from the CHN-GD16-05 (KY363868) isolate, was cloned into the prokaryotic expression vector pGEX-6p-1 at *Bam*HI and *Sal*I restriction sites. This configuration resulted in the fusion of the glutathione S-transferase (GST) protein to the N-terminus of the NS6 protein. In the *E. coli* expression system, the GST-NS6 fusion protein was designed to enhance the yield and immunogenicity of the recombinant NS6 protein. The correct construct was verified by sequencing and subsequently transformed into BL21 (DE3) competent cells for protein expression. 

A single colony was inoculated into 4 mL of Luria–Bertani (LB) medium supplemented with ampicillin and incubated at 37 °C with shaking at 220 rpm for 12 to 16 h. The overnight culture was then diluted 1:50 to 1:100 in 250 mL of 2× YT medium containing ampicillin. The cells were grown at 37 °C until the optical density at 600 nm (OD600) reached a value between 0.4 and 0.6. Protein expression was induced by the addition of isopropyl β-D-1-thiogalactopyranoside (IPTG) to a final concentration of 0.2 mM, and the culture was subsequently incubated at 37 °C for an additional 8 h. 

The cells were harvested by centrifugation, washed twice with sterile phosphate-buffered saline (PBS, pH 7.4), and lysed by sonication on ice for 15 min. The lysate was centrifuged to separate the soluble fraction (supernatant) from the insoluble components (pellet). Both fractions were resuspended in an equal volume of PBS and mixed with 5× sodium dodecyl sulfate (SDS) loading buffer. The samples were thoroughly mixed and denatured at 100 °C for 10 min before being analyzed by SDS-polyacrylamide gel electrophoresis (SDS-PAGE). The recombinant GST-NS6 fusion protein encoded by the pGEX-6p-1-NS6 construct was stained by 0.25 mol/L KCl solution and purified by gel extraction. The primers used for the cloning of the PDCoV *NS6* gene are listed in Table 1.

### 4.3. Generation of mAbs against PDCoV NS6 Protein

The purified GST-NS6 fusion protein was emulsified with an equal volume of Freund’s complete adjuvant (Sigma-Aldrich). Six- to eight-week-old female BALB/c mice were subcutaneously immunized at multiple sites with 200 μL of the emulsion containing 80 μg of the recombinant protein. Two weeks after the initial immunization, a second immunization was administered using the recombinant protein emulsified with an equal volume of Freund’s incomplete adjuvant (Sigma-Aldrich), following the same injection protocol as the initial immunization. A third immunization was conducted 14 days after the second, employing the same procedure as the second immunization. Seven to ten days after the third immunization, blood was collected from the orbital venous plexus to determine the antibody titer. The titer reached 1:1000, indicating a robust immunogenic response. A booster immunization was administered by intraperitoneal injection three weeks later with 200 μL containing 80 μg of the recombinant protein to further increase the antibody titer. Three days after the booster immunization, spleen cells from the immunized mice were fused with SP2/0 myeloma cells using polyethylene glycol 1500 (PEG-1500) to generate hybridoma cells.

### 4.4. Screening of PDCoV NS6 Protein Antigen

LLC-PK1 cells were seeded in a 96-well plate and cultured until they reached 80–90% confluence. After removal of the original culture medium, the cells were washed twice with PBS. The cells were then infected with PDCoV at a multiplicity of infection (MOI) of 0.1. Once pronounced cytopathic effects (CPE) were observed, but before extensive cell detachment occurred, the infected cells were fixed with 4% paraformaldehyde solution for 15 min. Subsequently, the cells were permeabilized with 0.2% Triton X-100 for 10 min and then blocked with 4% bovine serum albumin (BSA) in PBS at room temperature for 30 min. The hybridoma cell culture supernatants, presumed to contain antibodies against NS6, were added to each well as the primary antibody. Sera from immunized and non-immunized mice were included as positive and negative controls, respectively. The plate was incubated at 4 °C overnight. The next day, the cells were washed twice with PBS for two minutes each, and then incubated with a Dylight 488-conjugated goat anti-mouse IgG secondary antibody at 37 °C for one hour. After incubation with the secondary antibody, the cells were washed twice with PBS for two minutes each. The results were observed and captured under an inverted fluorescence microscope. 

### 4.5. Identification of Monoclonal Antibodies by Western Blot Analysis

LLC-PK1 cells were seeded in 6-well plates and cultured until reaching 80–90% confluence. The cells were then infected with PDCoV at an MOI of 0.1. The infected cells were harvested for protein extraction when the CPE reached approximately 50%. The extracted proteins were then separated by SDS-PAGE and transferred onto a polyvinylidene difluoride (PVDF) membrane. The membrane was then blocked with 5% non-fat milk in Tris-buffered saline containing 0.1% Tween-20 (TBST) for one hour at room temperature. The monoclonal antibody-containing ascites fluid, used as the primary antibody, was diluted 1:1000 in blocking buffer and incubated with the membrane overnight at 4 °C. Subsequently, the membrane was washed three times with TBST for 10 min each. The horseradish peroxidase (HRP)-conjugated goat anti-mouse IgG secondary antibody was diluted 1:10,000 in blocking buffer and incubated with the membrane for two hours at room temperature with gentle agitation. Subsequently, the membrane was washed three times with TBST, each for 10 min. Finally, the protein bands were visualized using enhanced chemiluminescence (ECL) detection reagents, and the membrane was imaged with the PVDF side facing up.

### 4.6. Mapping the Precise Epitope of PDCoV NS6 Protein

To precisely map the epitope on the PDCoV NS6 protein recognized by mAb 5-A11, we employed a strategy involving the expression of truncated NS6 proteins fused with enhanced green fluorescent protein (EGFP). The NS6 protein sequence, consisting of 94 amino acids, was divided into four overlapping peptide fragments (Figure 3): NS6-A (amino acids 1–40), NS6-B (amino acids 20–60), NS6-C (amino acids 41–79), and NS6-D (amino acids 60–94). Primers were designed to amplify and clone the fragments into the eukaryotic expression vector pEGFP-C3 between the *Xho* I and *Hind* III restriction sites. The recombinant plasmids were subjected to sequencing for verification and then transfected into HEK 293A cells. At twenty-four hours post-transfection, the expression of the EGFP-fused truncated NS6 proteins was observed under an inverted fluorescence microscope. Cells expressing high levels of the fusion proteins were harvested, and the reactivity of the expressed proteins with mAb 5-A11 was assessed by Western blot analysis.

To further narrow down the epitope, additional truncations were made from both the N-terminus and C-terminus of the reactive fragments. For N-terminal truncations, new upstream primers containing an *Xho* I site were designed, with the downstream primer remaining the same, targeting the *Mlu* I site in pEGFP-C3. Conversely, for C-terminal truncations, new downstream primers containing a *Hind* III site were designed, with the upstream primer remaining the same, targeting the *Nhe* I site in pEGFP-C3. The truncated fragments were cloned, expressed in HEK 293A cells, and analyzed by Western blot using the mAb 5-A11 as the primary antibody, in accordance with the previously described methodology. This iterative process was repeated until the truncated NS6 fragments were no longer recognized by mAb 5-A11, indicating that the epitope had been disrupted. The primers used to generate the truncated fragments are listed in Table 1. 

### 4.7. Prediction of NS6 Protein Structure and Epitope Distribution 

The theoretical three-dimensional structure of the NS6 protein from the PDCoV isolate CHN-GD16-05 (GenBank accession No. KY363868) was predicted using AlphaFold2 (version 2.3.2) [40]. The amino acid sequence of the NS6 protein was submitted to the ColabFold program (version 1.5.5) [41], which utilizes MMseqs2 for efficient homology search and employs the AlphaFold2 algorithm for accurate structure prediction. ColabFold simplifies the execution of AlphaFold2 and offers a user-friendly interface for submitting protein sequences and retrieving the predicted structures. The predicted three-dimensional structure of the PDCoV NS6 protein was visualized using PyMOL (open-source version 2.5.0) [42], an open-source molecular visualization system. The mAb binding epitope, spanning residues 71–80, on the NS6 protein of PDCoV, was accurately mapped and prominently displayed using PyMOL.

### 4.8. Epitope Sequence Similarity Analysis

Considering the homology between PDCoV and other coronaviruses, we conducted a sequence analysis of the epitope recognized by mAb 5-A11, focusing on PDCoV and δ coronaviruses. A total of 202 complete PDCoV genome sequences were retrieved from GenBank. Additionally, 18 δ coronavirus NS6 protein sequences homologous to the PDCoV NS6 protein were obtained from the InterPro database [43] (accession number: IPR044335) and the conserved domain database [44] (accession number: cd21629). The epitope and its flanking sequences of the NS6 protein were aligned with those of other selected coronavirus isolates using the MAFFT software [45] (version 7.525). Finally, the multiple sequence alignment results were visualized using the ggmsa package [46] (version 1.4.0) in R software [47] (version 4.4.0) to evaluate sequence conservation. The secondary structural elements of NS6 protein were analyzed using the online software ESPript (version 3.0) [48].

### 4.9. Cloning of the Variable Regions of mAb 5-A11 Heavy and Light Chains

The variable regions of the heavy chain (*VH*) and light chain (*VL*) of mAb 5-A11 were cloned using the following procedure. Total RNA was extracted from the hybridoma cell line secreting mAb 5-A11. The extracted total RNA was then reverse transcribed into cDNA. The *VH* and *VL* fragments were amplified by PCR using the cDNA as a template. The primers used to amplify the fragments of *VH* and *VL* are listed in Table 1. The amplified fragments were purified by agarose gel electrophoresis, recovered, and then ligated into the pMD19-T vector. The ligation products were transformed into competent *E. coli* DH5α cells. The plasmid DNA was extracted from the positive clones and sequenced to confirm the successful cloning of the *VH* and *VL* fragments.

## Figures and Tables

**Table 1:** Primers used in this study.

Primer Names	Primer Sequence ^1^ (5′–3′)		Size (bp)	Purpose of Primers
	Sense	Negative Sense		
NS6 protein	TGGGATCCATGTGCAACTGCCATCTGCA	GAGTCGACTTAATTTAATTCATCTTCAAGAATG	285	*NS6* gene cloning
NS6-A	CACCTCGAGATGTGCAACTGCCATCTGCAG	CACAAGCTTGTAAGCAAAACAGCGAGTTTT	120	Epitope analysis
NS6-B	CACCTCGAGATCAGAAGAGAGGATGTTCCA	CACAAGCTTAATTTTCCGAGGTAGTATGCT	123	Epitope analysis
NS6-C	CACCTCGAGAGTCTCGTGGTTCTTGCTAAT	CACAAGCTTAAAGTCTTTACCATATATGCT	117	Epitope analysis
NS6-D	CACCTCGAGATTCTTATCAATGGTGAGCCT	CACAAGCTTTTAATTTAATTCATCTTCAAG	108	Epitope analysis
NS6-D-1 (NS6-D-1-C80)	CACCTCGAGATTCTTATCAATGGTGAGCCT	CACAAGCTTGATAAAGTCTTTACCATATAT	63	Epitope analysis
NS6-D-1-C79	CACCTCGAGATTCTTATCAATGGTGAGCCT	CACAAGCTTAAAGTCTTTACCATATATGCT	60	Epitope analysis
NS6-D-1-N63	CACCTCGAGAATGGTGAGCCTTTACTGCTT	CACAAGCTTGATAAAGTCTTTACCATATAT	54	Epitope analysis
NS6-D-1-N70	CACCTCGAGGAATATGGTAGCATATATGGT	CACAAGCTTGATAAAGTCTTTACCATATAT	33	Epitope analysis
NS6-D-1-N71	CCCTCGAGTATGGTAGCATATATGGTAAAGA	CACAAGCTTGATAAAGTCTTTACCATATAT	30	Epitope analysis
pEGFP	ATCCGCTAGCGCTACCGGTCGCCA	CAATTTACGCGTTAAGATACATTGATG		Plasmid Verification
VL	ATGGAGACAGACACACTCCTGCTAT	GGATACAGTTGGTGCAGCATCAGCCCGTTT	420	Heavy chain cloning
VH	ATGGRATGSAGCTGKGTMATSCTCT	GGGSTGTYGTTTTGGCTGMRGAGACRGTGA	444	Light chain cloning

^1^ Restriction sites are shown as underlined.

## Data Availability

The data supporting the findings of this study are available within the article.
